# Supplementary material for: A Mixed-Methods Evaluation of a Post-COVID-Condition Rehabilitation and Recovery Intervention Delivered in a Football Club Community Trust
Source: Int J Environ Res Public Health. 2025 Nov 4;22(11):1672. doi: 10.3390/ijerph22111672 (PMC12652583; doi:10.3390/ijerph22111672)
Supplement: Supplementary file 1 [file ijerph-22-01672-s001.zip › ijerph-3794444-supplementary.pdf]

- Describe all your symptoms when you were diagnosed with long COVID.
- How did you function with daily activities before you got COVID-19, and how was it afterwards?
- How useful was the patient information provided to you at the time of your diagnosis?
- How did you feel when you were referred to our PCCRRP?
- How did you feel about the PCCRRP being delivered by a FCCT?
- How did you find traveling to the PCCRRP? Was it a positive or a barrier?
- Since you completed our PCCRRP, how were your symptoms after? What did you feel like after the 12 weeks?
- How do you feel mentally and physically on completion of the program, and how has it impacted your daily life?
- Did you feel supported by our staff on the PCCRRP?
- Would you recommend the PCCRRP to other people?
- Are there any suggestions for improvements for the PCCRRP?
